# Gastro-Protective Effects of *Albizia anthelmintica* Leaf Extract on Indomethacin-Induced Gastric Ulcer in Wistar Rats: In Silico and In Vivo Studies

**DOI:** 10.3390/antiox10020176

**Published:** 2021-01-26

**Authors:** Mohamed Nabil, Mohamed A. El Raey, Walied Abdo, Mohamed A. O. Abdelfattah, Assem M. El-Shazly, Mansour Sobeh, Mona F. Mahmoud

**Affiliations:** 1 Pharmaceutical and Fermentation Industries Development Center (PFIDC), City of Scientific Research and Technological Applications (SRTA-City), New Borg El-Arab 21934, Egypt; mnm01120139193@gmail.com; 2 Pharmacology Department, Faculty of Pharmacy, Deraya University, New Mina 61768, Egypt; 3 Department of Phytochemistry and Plant Systematics, Pharmaceutical Division, National Research Centre, Dokki, Cairo 12622, Egypt; elraiy@gmail.com; 4 Department of Pathology, Faculty of Veterinary Medicine, Kafrelsheikh University, Kafr El Sheikh 33516, Egypt; waliedsobhy40@gmail.com; 5 College of Engineering and Technology, American University of the Middle East, Kuwait; mohamed.abdelmoety@aum.edu.kw; 6 Department of Pharmacognosy, Faculty of Pharmacy, Zagazig University, Zagazig 44519, Egypt; assemels2002@yahoo.co.uk; 7 AgroBioSciences Research Department, Mohammed VI Polytechnic University, Lot 660–Hay MoulayRachid, Ben-Guerir 43150, Morocco; 8 Department of Pharmacology and Toxicology, Faculty of Pharmacy, Zagazig University, Zagazig 44519, Egypt

**Keywords:** *Albizia anthelmintica*, gastric ulcer, indomethacin, flavonoids

## Abstract

We have previously reported that the leaf extract of *Albizia anthelmintica* exhibited substantial antioxidant, anti-inflammatory, analgesic, and antipyretic properties in vivo. We also comprehensively characterized the active phytoconstituents and found several flavonoids and galloyl glucosides derivatives. In the current work, we explored the gastroprotective effects of the leaf extract in an indomethacin-induced ulcer model and the mechanisms involved. The rats being pretreated with the tested extract (100 and 200 mg kg^−1^) significantly prevented gastric lesions by 87.4% and 92.3%, respectively, and they had no structural derangements in the gastric mucosa. The extract significantly reduced the elevated levels of IKκB, NF-κB, TNF-α, IL-6, iNOS, and lipid peroxidation; increased the reduced level of glutathione peroxidase (GPx) activity; and reduced glutathione (GSH) in the indomethacin-induced ulcer model. The protective activities of the extract were similar in most aspects to those exerted by the known anti-ulcer drug famotidine. These activities might be attributed to the anti-inflammatory and antioxidant activities, and the reduction of iNOS levels. In conclusion, *Albizia anthelmintica* is a potential candidate for management of gastric ulcers with antioxidant properties.

## 1. Introduction

Peptic ulcer disease (PUD) is a common pathological condition of the gastrointestinal tract, and although its annual incidence rate—estimated to range from 0.1 to 0.19%—has been declining recently, the disease is still irritating to multiple categories of individuals, especially the elderly [1,2]. In PUD, the protective mechanisms of the gastrointestinal mucosa, including the bicarbonate and mucous secretions, are largely overwhelmed by the mucosal damage induced by the gastric acid and pepsin hyper-secretions. A burning, night-awakening epigastric pain that is usually relieved by either ingesting food or taking an antacid is the perspicuous symptom of the disease [3].

PUD is caused mainly by infection with the Gram-negative *Helicobacter pylori* spiral rods, the long-term administration of the non-steroidal anti-inflammatory drugs (NSAIDs), excessive consumption of alcohol, or continuous stress [4]. The use of NSAIDs induces primary mucosal irritation by virtue of the prominent acidic nature of these drugs and secondary or indirect injury to the gastric mucosa through blocking the synthesis of the gastro-protective thromboxane and prostaglandins [5]. Apart from that, all the former noxious factors, including NSAIDs, can aid the ulcer formation through generating different types of reactive oxygen species (ROS), which stimulate the production of some pro-inflammatory cytokines, such as nuclear factor kappa (NF-κB) and tumor necrosis factor-α (TNF-α) [6].

Left ignored or not properly treated, PUD complications may result in gastroduodenal perforation, hemorrhage, and obstruction [7]. Treatment usually depends on the use of anti-secretory agents, including H2-receptors blockers and proton pump inhibitors (PPIs), along with some antibiotics such as clarithromycin and/or metronidazole in case *H. pylori* infection is proven active by laboratory testing [8,9]. 

However, the long-term use of PPIs is associated with some serious adverse effects, such as fractures, kidney disease, and higher susceptibility to certain infections, and calcium, magnesium, and vitamin B12 deficiencies [10]. The H2-blocker ranitidine was phased out of market in many countries over the detection of above-the-limit levels of the carcinogenic contaminant *N*-nitrosodimethylamine (NDMA). In this regard, natural secondary plant metabolites could offer safer alternatives. Discovery of new drug candidates processing anti-secretory and antioxidant properties could have an augmented ripple effect in treating PUD [11,12].

The worm-cure *Albizia*, *Albizia anthelmintica*, belongs to the genus *Albizia*, family Fabaceae. The plant is widely used in traditional medicine. We recently characterized the plant’s secondary metabolites from trees grown in Egypt. The analysis revealed 40 compounds, among which quercetin-*O*-galloyl-glucoside dominated. The extract displayed substantial antioxidant, anti-inflammatory, analgesic, and antipyretic properties in vivo. Our results were confirmed utilizing in vitro enzyme inhibitory assays (COX-1, COX-2, and 5-LOX) and molecular docking studies [13]. 

In the current study, we explored the protective effects of the leaf extract against indomethacin-induced gastric ulcers in rats. We also discussed several oxidative stress markers, inflammatory cytokines, and nitrosative stress markers’ levels for the biochemical mechanism. Additionally, we virtually studied the possible interactions of the individual components against two enzymes that are involved in ulcers (histidine decarboxylase and gastric proton pump).

## 2. Materials and Methods

### 2.1. Drugs and Chemicals

Indomethacin was purchased from Nile company for pharmaceuticals, Cairo, Egypt; Famotidine, was obtained from Amoun pharmaceutical company, Salam, Cairo, Egypt. Thiopental sodium was purchased from EIPICO pharmaceutical Company, 10th of Ramadan city, Egypt. All other chemicals were analytical grade and purchased from Sigma (St. Luis, MO, USA).

### 2.2. Plant Material and Extraction

The leaves were collected from El-Zohria Garden, Giza, Egypt. The leaves were dried, ground, and extracted using methanol (3 × 3 L) at ambient temperature. After combining the extracts, they were concentrated under reduced pressure and then freeze-dried to provide an extraction yield of 8.6% [13].

### 2.3. Ethical Statement

All experimental procedures conformed to the Guide for the Care and Use of Laboratory Animals published by US National Institutes of Health and with the approval of the Animal Ethics Committee of Zagazig University (ZU-IACUC) under the protocol approval number ZU-IACUC/3/F/73 /2020.

### 2.4. Animals

Male Wistar rats, weight range 180 ± 20 g (Faculty of Veterinary medicine, Zagazig, Egypt), were used for the experiments. Rats were placed in five cages with wood shavings as bedding. The rats were housed in normal laboratory conditions (Temperature 22 °C and relative humidity 50–55% with 12 h light/dark cycle) for one week before starting the experiments. The rats had free access to standard rodent food and water ad libitum. 

### 2.5. Experimental Design

**Scheme 1:**
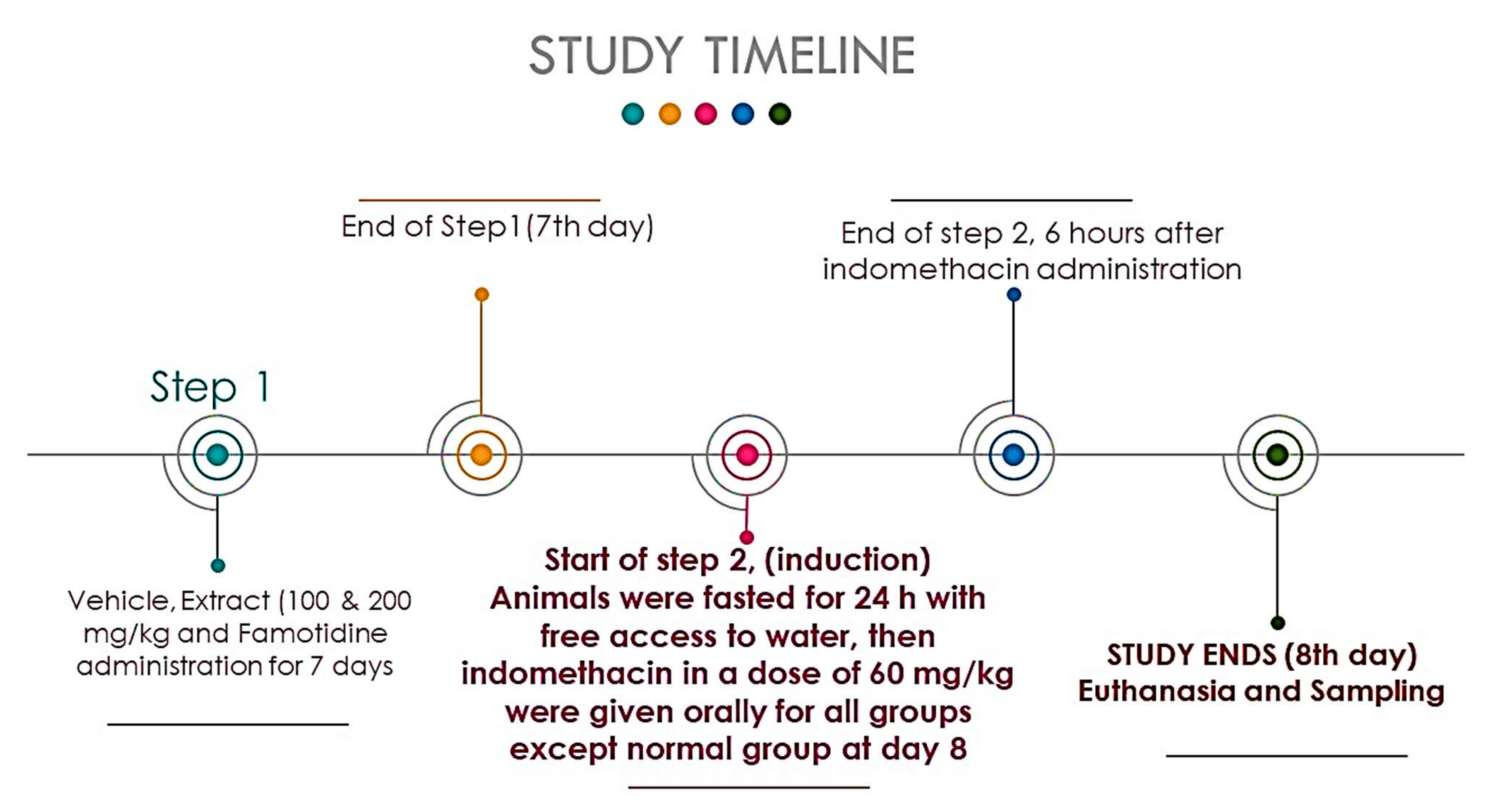
Time course of experimental schedule. Control, ulcer, famotidine, and *Albizia anthelmintica* extract groups.

The gastric ulcer was induced using indomethacin (single dose, 60 mg/kg body weight) according to a method described previously [14]. Thirty rats were divided into 5 groups (6 rats/ group). Group 1 received vehicle (5% tween 80) orally for 8 days (2 mL/kg/day). Group 2 received vehicle (5% tween 80) orally for 8 days, and then indomethacin on the 8th day. Group 3 received famotidine 10 mg/kg suspended in 2 mL of 5% tween 80 orally for 8 consecutive days followed by indomethacin at the 8th day. Groups 4 and 5 received 100 and 200 mg/kg of *A. anthelmintica* extract, respectively, for 8 consecutive days, and then indomethacin on the 8th day. Animals in groups 2–5 were fasted for 24 h but had free access to water on the last day of the experiment. Then, they received 60 mg/kg indomethacin suspended in 5% tween 80 by oral gavage for ulcer induction, as shown in Scheme 1.

### 2.6. Blood and Tissue Sampling

Six hours after administration of indomethacin, animals were anaesthetized by thiopental sodium (50 mg/kg); blood was collected from orbital plexus and centrifuged (4000 rpm, 4 °C, 15 min) to obtain the serum, which was stored at −80 °C. The stomachs of the rats were collected and opened at the greater curvature to collect the gastric contents. The latter were centrifuged and used to determine the gastric secretion parameters, such as acidity and pH. Gastric mucosa was investigated for estimation of ulceration degree, which was presented as ulcer index and percentage of ulcer inhibition. A part of the stomach of each rat was carefully excised, weighed, and fixed in 10% formalin-saline for histopathological studies. The other part was snap frozen in liquid nitrogen and kept at −80 to be used for subsequent tissue analyses.

### 2.7. Assessment of Gross Mucosal Damage

The stomach of each rat was washed with ice-cold saline and blotted dry between 2 filter papers. Then, the cleaned stomach was stretched and pinned on a corkboard, and digital images were taken for the stomachs to evaluate mucosal damage. The images were analyzed with ImageJ software (Wayne Rasband, MD, USA); the areas of ulcerations were measured and the ulceration ratio was calculated according to the methodology outlined by Szabo and Hollander (Szabo and Hollander 1989), where the ulcer index (U.I.) of each animal was calculated according to the following formula: 

U.I. = [ulcerated area/total stomach area] × 100, and the percentage of inhibition against ulceration was determined using the formula: % ulcer inhibition = [U.I. in ulcer group − U.I. in test group] × 100/U.I. in ulcer group.

### 2.8. Measurement of Gastric Acidity

The excised stomach was incised through the line of the greater curvature and rinsed in 5 mL of ice-cold distilled water, and the resultant wash was drained into centrifuge tubes, and centrifuged for 10 min at 3000 rpm. The supernatant was used to measure the pH of gastric juice using a pH meter (Mettler Toledo™ FiveEasy Plus™ FEP20 pH Meter, Fisher Scientific AS, Oslo, Norway).

### 2.9. Assessment of Gastric Tissue Oxidative Stress Markers

One gram of stomach tissue was rinsed for two minutes in an ice-cold solution of phosphate buffered saline (PBS) (pH 7.4, containing 0.16 mg/mL heparin). The tissue was homogenized in 10 mL of ice-cold 50 mM potassium phosphate buffer (pH 7.5, containing 1% protease inhibitor cocktail, Boster Biological Technology, Pleasanton, CA, USA, catalogue number: AR1182). The homogenate was centrifuged for 15 min at 4000 rpm at 4 °C. The total protein contents of the supernatant were measured spectrophotometrically using the Bradford method [15]. The level of lipid peroxidation products represented by malondialdehyde (MDA) in the tissue supernatant, as a marker of oxidative stress, was measured according to the colorimetric method of Tappel and Zalkin [16] and expressed as µmol/mg protein. The antioxidant markers were evaluated through the measurement of the reduced glutathione (GSH) level according to the method described by Ellman [17] and expressed as µmol/mg protein; and the glutathione peroxidase (GPx) activity was measured as described in the method of Paglia and Valentine [18] and expressed as U/mg protein.

### 2.10. Determination of Gastric Tissue Inflammatory Markers IL-6 and TNF-α

The protein concentration in gastric tissue supernatant was normalized at the ratio of 1 mg of protein per 1 mL of lysate solution with the fresh addition of a protease inhibitor cocktail before proceeding to ELISA measurement. The levels of interleukin (IL)-6 and tumor necrosis factor-alpha (TNF-α) were measured in the tissue lysate using rat ELISA kits supplied by Sigma-Aldrich, Missouri, United States (catalogue number: RAB0480 and RAB0311, respectively) according to the manufacturer’s instructions.

### 2.11. Determination of Gastric Tissue Protein Expression of NF-κB and IKκB 

Gastric tissue was homogenized in ice-cold Nonidet-P40 (NP40) buffer with the addition of 1% protease inhibitor. Then, homogenate was incubated at 4 °C for 2 h on shaker before it was centrifuged at 4 °C, 13,000 rpm for 20 min. Protein content of the sample was determined using the Bradford assay method [15]. Then, it was denatured by boiling for 5 min in 2× Laemmli buffer [19], and equal concentrations of the pooled proteins were loaded into 10% sodium dodecyl sulfate-poly-acrylamide gel (SDS-PAGE) and transferred onto nitrocellulose membranes using Trans-Blot^®^ semi-dry transfer cell (Bio- Rad, Hercules, CA, USA). They were blocked in 1× Tris-buffered saline/0.1% Tween 20 (TBST) with 5% bovine serum albumin (BSA) and incubated with the primary antibody in 1× TBST/3% BSA at 4 °C. The membrane was washed six times (10 min) in 1× TBST and incubated with KPL alkaline phosphatase conjugated secondary antibodies goat anti-rabbit and goat anti-mouse for 1 h and then with nitro blue tetrazolium/5-bromo-4-chloro-3-indolyl-phosphate (NBT/BCIP) solution (Thermo Fisher Scientific, Waltham, MA, USA) for visualization of the protein bands. All expressed proteins were normalized to β-actin content and were measured with Image Studio Lite Software (LI- COR Biotechnology, Lincoln, NE, USA) [20]. Primary rabbit polyclonal antibodies against inhibitor of nuclear factor kappa-B kinase subunit beta (IKK-β, catalogue number: PA2036-1) and rabbit monoclonal antibodies against β-actin (catalogue number: M01263) were purchased from Boster Biological Technology (Pleasanton, CA, USA) and diluted at 1:1000 and 1:5000, respectively. Primary mouse monoclonal antibodies against nuclear factor kappa-B (NF-κB) p65 (catalogue number: sc-8008) were purchased from Santa Cruz Biotechnology (Dallas, TX, USA) and diluted at 1:200. The secondary antibodies goat anti-rabbit (catalogue number: 5220–0308) and goat anti-mouse (catalogue number: 5220–0310) were purchased from SeraCare, Milford, MA, USA and diluted 1:10,000.

### 2.12. Microscopic Assessment of Gastric Ulcers

The stomach tissues were fixed in 10% neutral buffered formalin for one day, followed by dehydration and embedding in paraffin wax, before they were transversally sectioned to 5 μm thick by sledge microtome. For detection of microscopic gastric injury, hematoxylin and eosin staining was used for histopathological examination under the light microscope [21]. Histopathological changes were evaluated according to Ortac et al. [22] by an experienced pathologist blinded to treatments. Gastric structural derangement was determined on a 0–4 scale, and each tissue section was examined for hemorrhagic damage (0–4), mucosal edema (0–4), presence of inflammatory cells infiltration (0–3), and epithelial cell loss (0–3). Periodic acid-Schiff (PAS) stain was used to detect mucosal glycoprotein fabrication [23]. 

### 2.13. Immunohistochemical Evaluation of Gastric Inducible Nitric Oxide Synthase (iNOS)

The sections (5 µm) were dewaxed, hydrated, and immersed in an antigen retrieval medium (EDTA solution, PH 8). Then, they were treated with hydrogen peroxide 0.3% and protein block and incubated with rabbit anti-iNOS polyclonal antibody (Invitrogen, PA1-036, USA) diluted 1:20 dilution for 1 h at room temperature. Sections were washed three times with PBS and incubated with anti-rabbit IgG secondary antibodies (EnVision + System HRP; Dako, Glostrup, Denmark) for 30 min at room temperature (EnVision + System HRP; Dako). Subsequently, they were visualized with di-aminobenzidine commercial kits (Liquid DAB + Substrate Chromogen System; Dako), and finally counterstained with Mayer’s hematoxylin. As a negative control procedure, the primary antibody was replaced by normal mouse serum. The labeling index of iNOS was expressed as a percentage of positive area per total area in 8 high power fields [24]. 

### 2.14. Molecular Modelling

The crystal structures of histidine decarboxylase (PDB ID 4E1O) and gastric proton pump (PDB ID 5YLV) were downloaded from the protein data bank (www.pdb.org). The compounds were downloaded from Pubchem (https://pubchem.ncbi.nlm.nih.gov/) as SDF files. The docking studies were carried out by molecular operating environment (MOE) software, 2013.08 (Chemical Computing Group Inc.; Montreal, QC, Canada, H3A 2R7, 2016), as detailed in El Hawary et al. [25].

### 2.15. Statistical Analysis

Results are expressed as mean ± SE. Multiple comparisons were done utilizing one-way ANOVA followed by Tukey–Kramer tests for post hoc analyses. All statistical analyses were conducted by GraphPad Prism (GraphPad software Inc., La Jolla, CA, USA). Histopathological scores’ statistical variation among groups was tested by Kruskal–Wallis test followed by Dunn’s multiple comparisons test. *p* value ˂ 0.05 was considered statistically significant.

## 3. Results

### 3.1. Compound Isolation and Phytochemical Profiling

**Figure 1:**
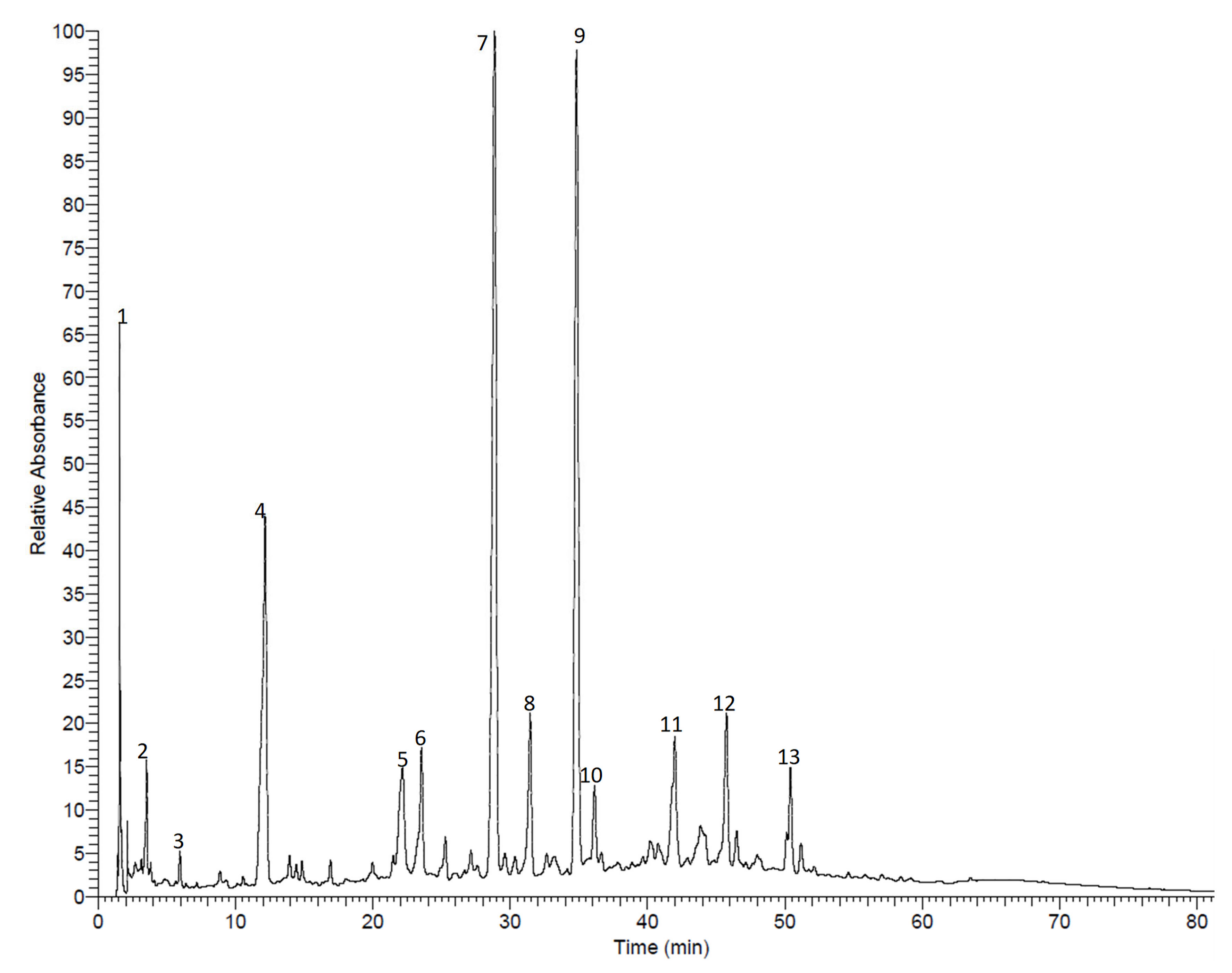
HPLC-PDA chromatogram of the methanol extract from *A. anthelmintica* leaf. (**1**) 3-Galloyl-glucose, (**2**) 2-galloyl-glucose, (**3**) gallic acid; (**4**) methyl gallate, (**5**) hydroxybenzyl-*o*-galloyl-glucose, (**6**) coumaroyl-*o*-galloyl-glucose, (**7**) quercetin-*o*-galloyl-glucoside, (**8**) quercetin glucoside, (**9**) kaempferol galloyl-glucoside, (**10**) kaempferol galactoside, (**11**) quercetin caffeoyl-glucoside, (**12**) quercetin coumaroyl-glucoside, (**13**) kaempferol coumaroyl-glucoside. The compounds were identified based on their molecular weights and mass fragmentation patterns, as reported before [13].

In our previous work, LC-MS profiling of phytoconstituents of the extract revealed 40 compounds, mainly flavonoids and galloyl glucosides derivatives [13]. In this study, we isolated two compounds, namely, gallic acid and methylgallate, using paper chromatography. The compounds were identified according their retention factor values, molecular weights, and mass fragmentation patterns, and were confirmed using authentic compounds. Figure 1 represents the HPLC-PDA chromatogram of the extract.

### 3.2. Effect of A. anthelmintica Extract on Ulcer Index of Indomethacin-Treated Rats

**Figure 2:**
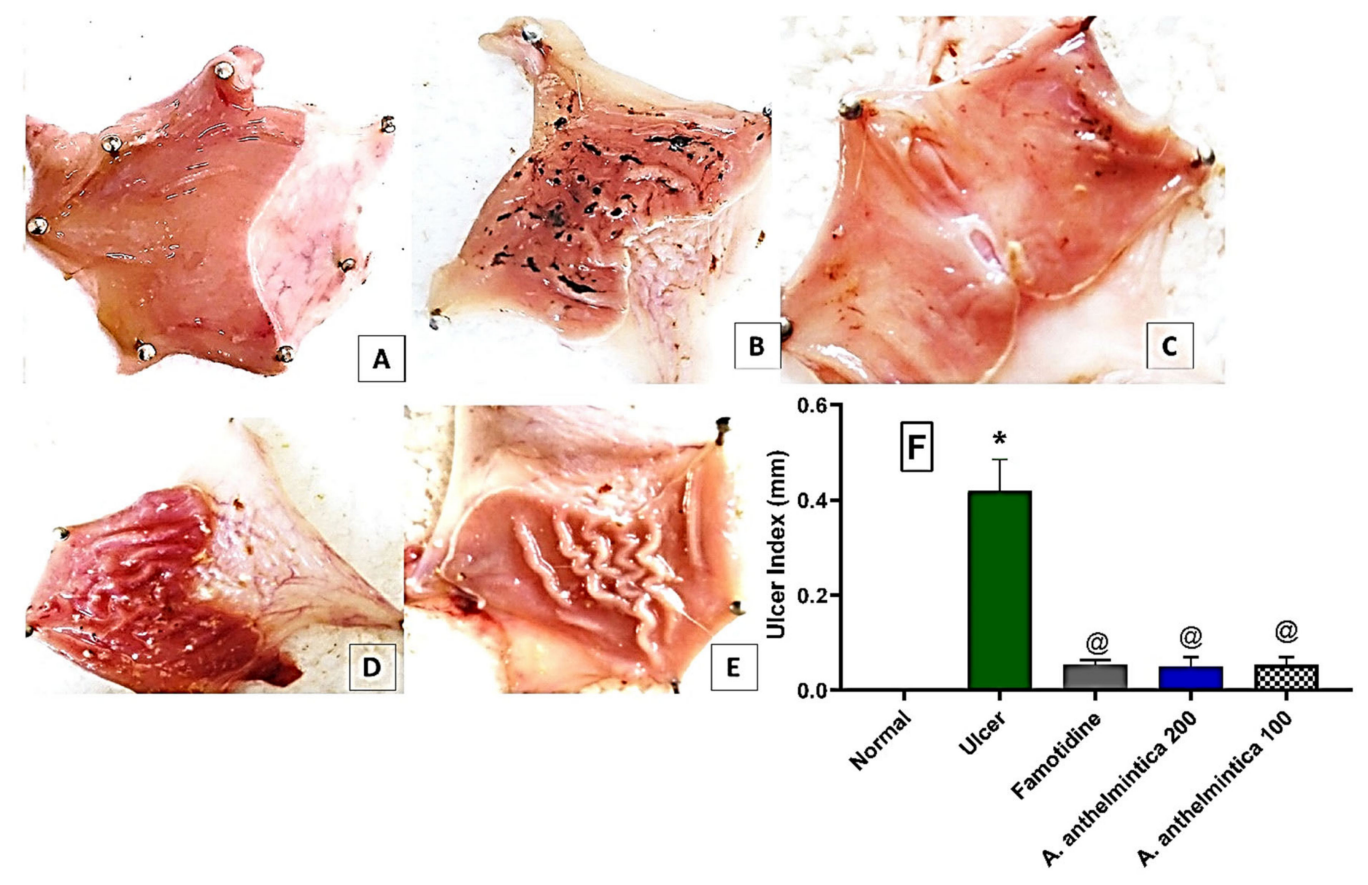
Representative rat stomach photomacrographs that were cut along the greater curvature. (**A**) Control, (**B**) indomethacin, (**C**) reference drug (famotidine), (**D**) *A. anthelmintica* extract (200 mg/kg), (**E**) *A. anthelmintica* extract (100 mg/kg) representing the substantial existence of many circular and linear gastric ulcers in the indomethacin group, which were significantly reduced by the reference drug (famotidine) and the two tested doses of *A. anthelmintica* extract. (**F**) The effects of indomethacin without or with pre-treatments of famotidine, and the tested extract (*A. anthelmintica*, 200, 100 mg/kg) on the gastric ulcer index in rats. Statistical analyses were carried out utilizing one-way ANOVA followed by Tukey’s post hoc test, *n* = 5–6, mean ± SEM. * Significantly different from control group at *p* < 0.05. ^@^ Significantly different from indomethacin alone (ulcer) group at *p* < 0.05.

Macroscopic examination of the stomachs isolated from indomethacin treated rats showed many circular and longitudinal ulcers. Photomacrographs of the stomachs isolated from different treatment groups showed few ulcers, indicating preventive effects for the reference drug (Famotidine) and the tested extract. The extract at the two dose levels exhibited similar effects to famotidine (Figure 2). The tested doses of the extract (100 and 200 mg/Kg) significantly reduced the ulcer index compared to indomethacin group (*p* < 0.05), with similar effects to those reported from famotidine. The preventive ratio was 92.3% for the extract (200 mg/kg) and 87.4% for the extract (100 mg/kg) and famotidine.

### 3.3. Effect of A. anthelmintica Extract on Gastric Damage Alterations Induced by Indomethacin in Rats

**Figure 3:**
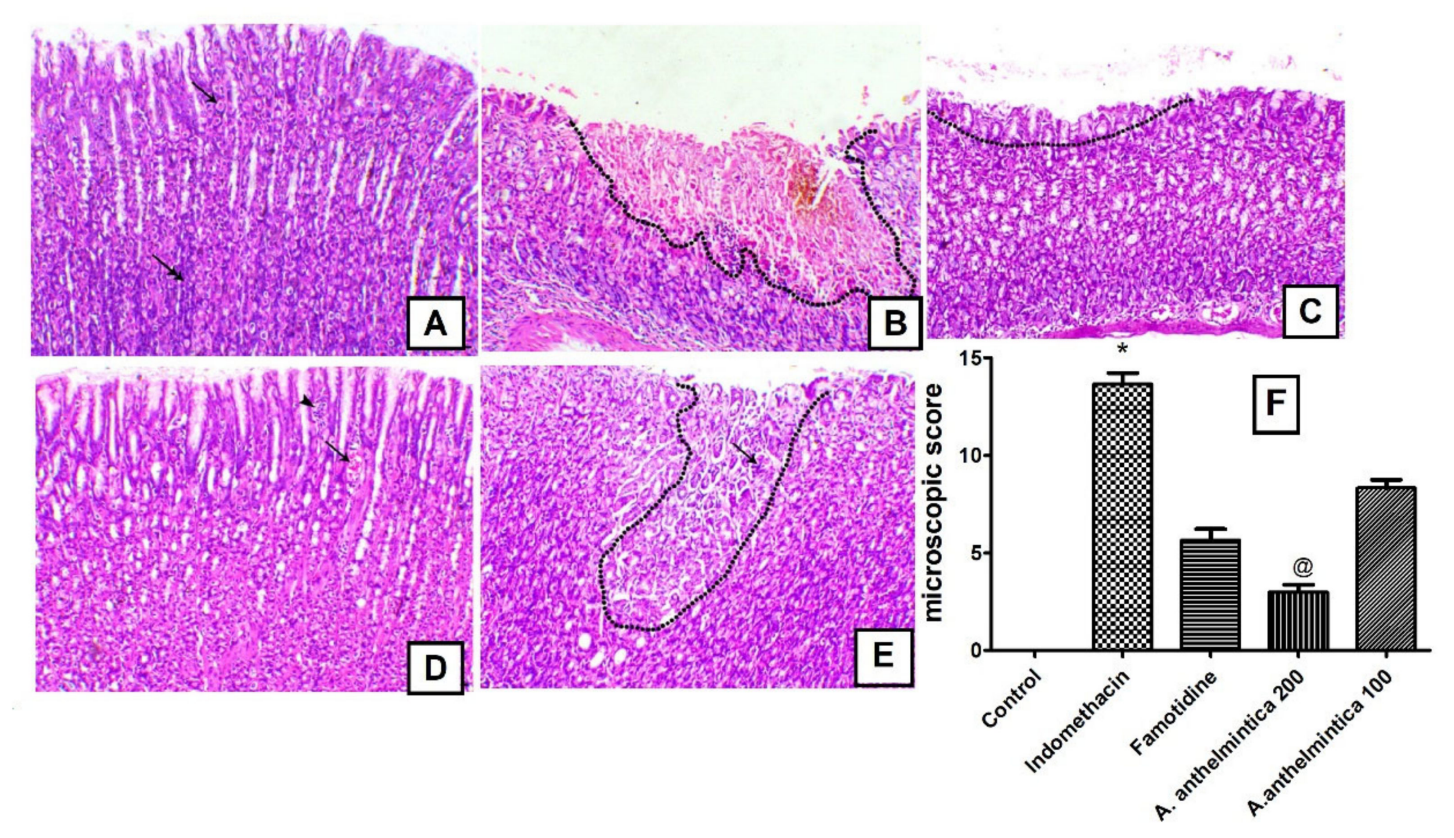
A photomicrograph of histopathological changes: (**A**) Control with normal gastric mucosa (arrow indicates normal parietal cells and double arrow indicates normal surface mucous cells); (**B**) indomethacin group showing severe degree of coagulative necrotic changes within the gastric mucosa (area above the dotted line) accompanied with deposition of acid hematin (arrow) and inflammatory cell infiltration (arrowheads); (**C**) famotidine-treated group showing marked decreases in the degenerative and necrotic changes within the gastric mucosa (area above the dotted line); (**D**) *A. anthelmintica* extract (200 mg/kg)-treated group showing a marked decrease in the degenerative changes within gastric glands (arrow indicates mild congestion of the gastric capillaries) and few lymphocytes within the surface mucosal lining (arrowhead); (**E**) *A. anthelmintica* extract (100 mg/kg)-treated group showing foci of degenerative gastric mucosa (area above the dotted line) revealing few necrotic gastric glands (arrow)), H&E, 100×, bar = 100 µm. (**F**) Microscopic scores in gastric tissues of rats of different experimental groups. Values are mean ± SEM from 6 samples using Kruskal–Wallis followed by Dunn’s post hoc test. * *p* < 0.05, compared with control; ^@^ *p* < 0.05, compared with indomethacin.

As shown in Table 1 and Figure 3, histopathological examination of the stomachs of different rat groups revealed that administration of indomethacin caused remarkable gastric damage, as evidenced by the existence of severe degrees of coagulative necrotic changes within the gastric mucosa accompanied by the deposition of acid hematin and the infiltration of inflammatory cells. Pre-treatment with famotidine markedly mitigated the pathological changes in the stomachs, as manifested by the marked decrease of the degenerative and necrotic changes within the gastric mucosa. Pre-treatment of rats with the extract at a high dose level (200 mg/kg) resulted in a marked reduction of the degenerative changes within gastric glands by inhibition of gastric inflammation within the surface mucosal lining. On the other hand, the low dose level of the extract (100 mg/kg) provided a lesser improvement than both the high dose and famotidine, as manifested by the presence of foci of degenerative gastric mucosa, revealing a few necrotic gastric glands. As shown in Table 1 and Figure 3, statistical analysis showed that indomethacin significantly increased gastric necrosis, edema, hemorrhage, and inflammation compared with the control group (*p* < 0.05). Only the high dose of the extract significantly reduced the aforementioned changes compared with the indomethacin group (*p* < 0.05).

### 3.4. Effect of A. anthelmintica Extract on Gastric Acidity Alterations Induced by Indomethacin in Rats

**Figure 4:**
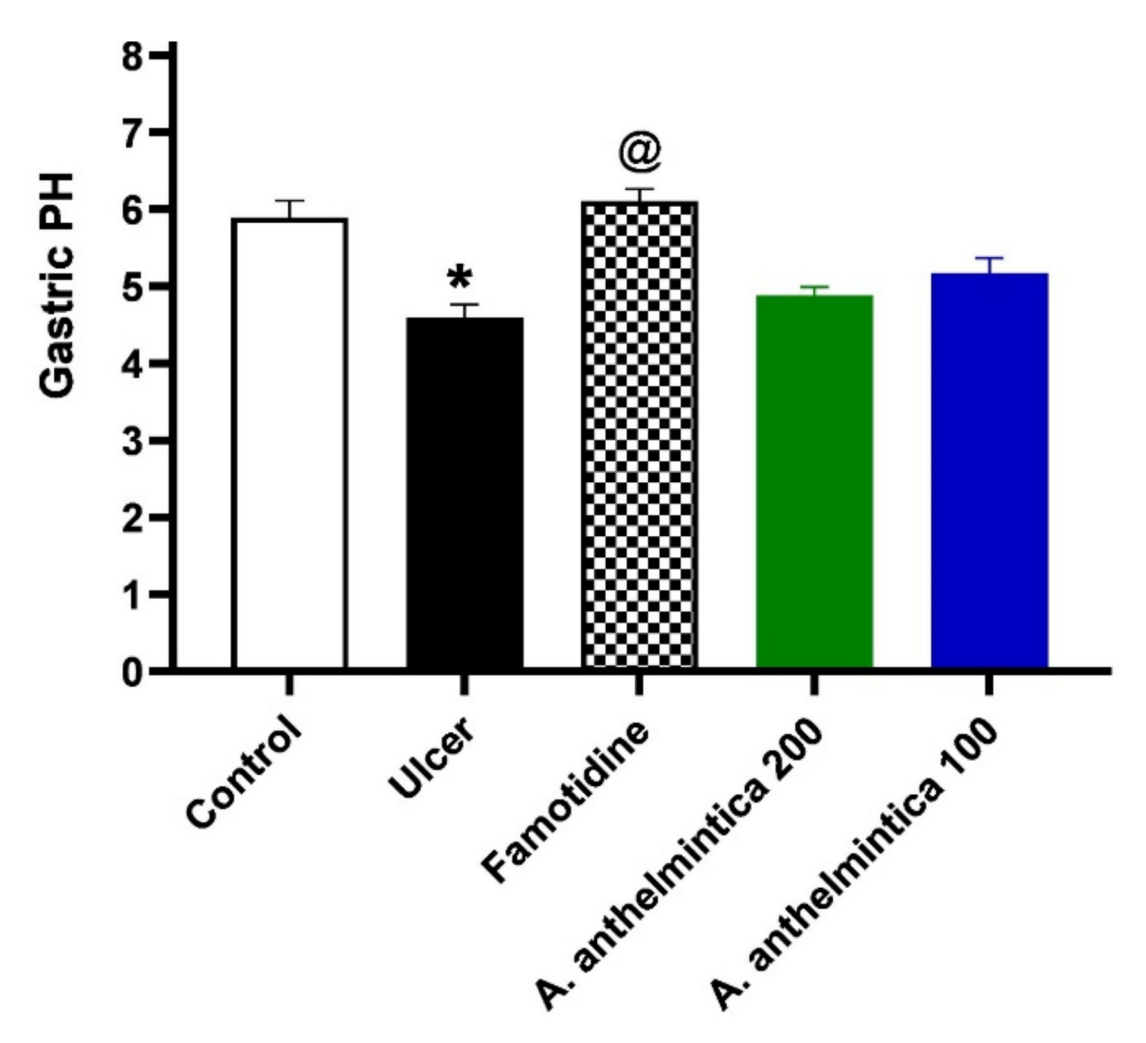
Effects of indomethacin alone or with oral pre-treatments of famotidine, and *A. anthelmintica* extract (200, 100 mg/kg) on the gastric pH in rats. Statistical analyses were conducted utilizing one-way ANOVA followed by Tukey’s post hoc test, *n* = 6, mean ± SEM. * Significantly different from control group at *p* < 0.05. ^@^ Significantly different from indomethacin alone group at *p* < 0.05.

Gastric acidity was increased, manifested by the reduction in gastric pH in indomethacin-treated rats compared with control group (Figure 4, *p* < 0.05). Pre-treatment with famotidine decreased gastric acidity compared to the indomethacin group (*p* < 0.05). Both dose levels of the extract did not affect the gastric acidity significantly compared with indomethacin-alone group (Figure 4, *p* ˃ 0.05).

### 3.5. Effects of A. anthelmintica Extract on Gastric Mucin Alteration Induced by Indomethacin in Rats

**Figure 5:**
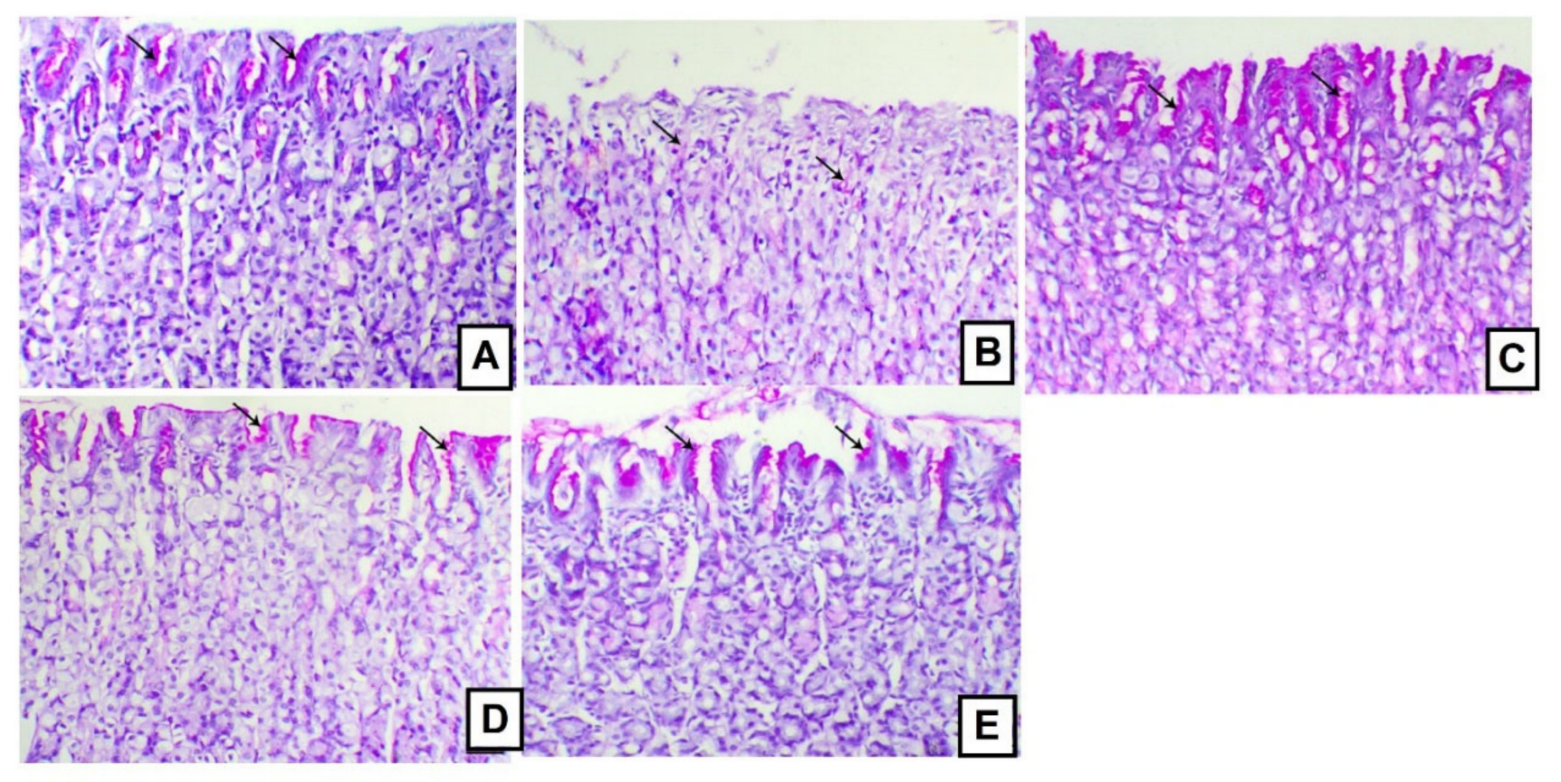
A photomicrograph of a stomach. (**A**) Control group displaying normal glycoproteins secretion within the mucosal lining of the gastric pits of the gastric glands (arrows); (**B**) indomethacin group showing a marked decrease of glycoproteins within gastric mucosa (arrows); (**C**) famotidine-treated group showing a marked increase of glycoproteins within the gastric glands (arrows); (**D**) *A. anthelmintica* extract (200 mg/kg)-treated group showing an increase of glycoprotein content within the gastric glands (arrows); (**E**) *A. anthelmintica* extract (100 mg/kg)-treated group showing an increase of glycoproteins within the covering mucosa of the gastric glands (arrows), PAS stain, 200×, bar = 50 µm.

Periodic acid–Schiff (PAS) histochemical staining is utilized to reveal the existence of macromolecules such as glycoproteins, proteoglycans, and glycogen that are typically found in the mucus to protect the gastric mucosa. PAS staining of the stomach from animals treated with indomethacin was decreased, indicating a marked decrease of glycoprotein content within the gastric mucosa when compared to the control group; see Figure 5B. Microscopic examination of the PAS staining in the gastric mucosa from rats treated with famotidine and high and low doses of the extract showed marked increases in gastric glycoprotein content, revealing the healing effects of both famotidine and the extract; see Figure 5C–E.

### 3.6. Effects of A. anthelmintica Extract on Changes in Oxidative Stress Markers Induced by Indomethacin in Rat Gastric Mucosa

**Figure 6:**
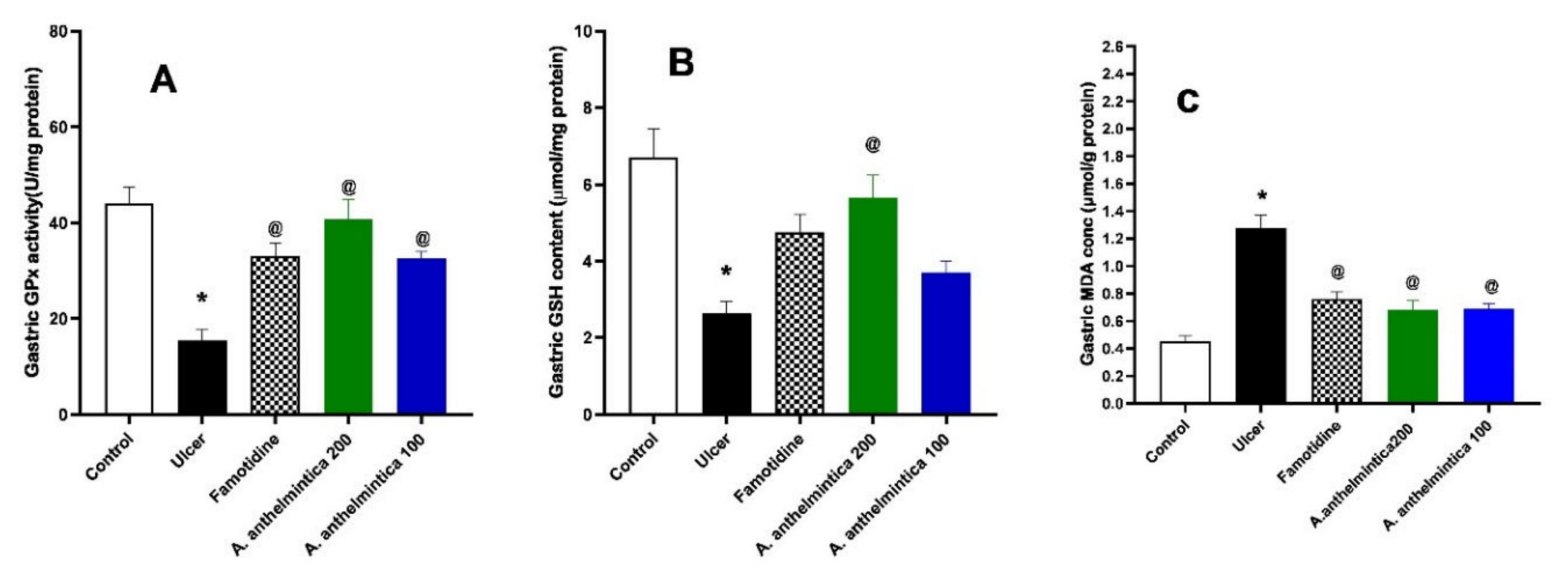
Effects of indomethacin alone and with oral administration of famotidine, or *A. anthelmintica* extract (200, 100 mg/kg) on (**A**) gastric glutathione peroxidase activity (GPx), (**B**) gastric reduced glutathione (GSH), and (**C**) lipid peroxidation product (malondialdehyde) in rats. Statistical analyses were carried out by one-way ANOVA followed by Tukey’s post hoc test, *n* = 6, mean ± SEM. * Significantly different from control group at *p* < 0.05. ^@^ Significantly different from indomethacin alone group at *p* < 0.05.

To identify the gastroprotective mechanism of the extract, we further analyzed oxidative stress markers of the gastric mucosa and found that indomethacin decreased both gastric glutathione peroxidase activity by 65% and glutathione content by 60.6% compared to the control group (*p* < 0.05). Pre-treatment of rats with famotidine or the extract at a high or low dose increased gastric glutathione peroxidase activity—114%, 163.6%, and 110.8% respectively (*p* < 0.05, Figure 6A). However, only the extract at the high dose exerted a significant effect on the glutathione gastric content (*p* < 0.05, Figure 6B). On the other hand, lipid peroxidation represented by MDA was elevated in the indomethacin group by 184.4% (*p* < 0.05), compared to the control group. Both famotidine and the extract at the two dose levels decreased gastric MDA level by 40.6%, 46.9%, and 46.1% (*p* < 0.05) respectively compared to the indomethacin group (Figure 6C). 

### 3.7. Effect of Albizia anthelmintica on Inflammatory Changes Induced by Indomethacin in Rat Gastric Mucosa

**Figure 7:**
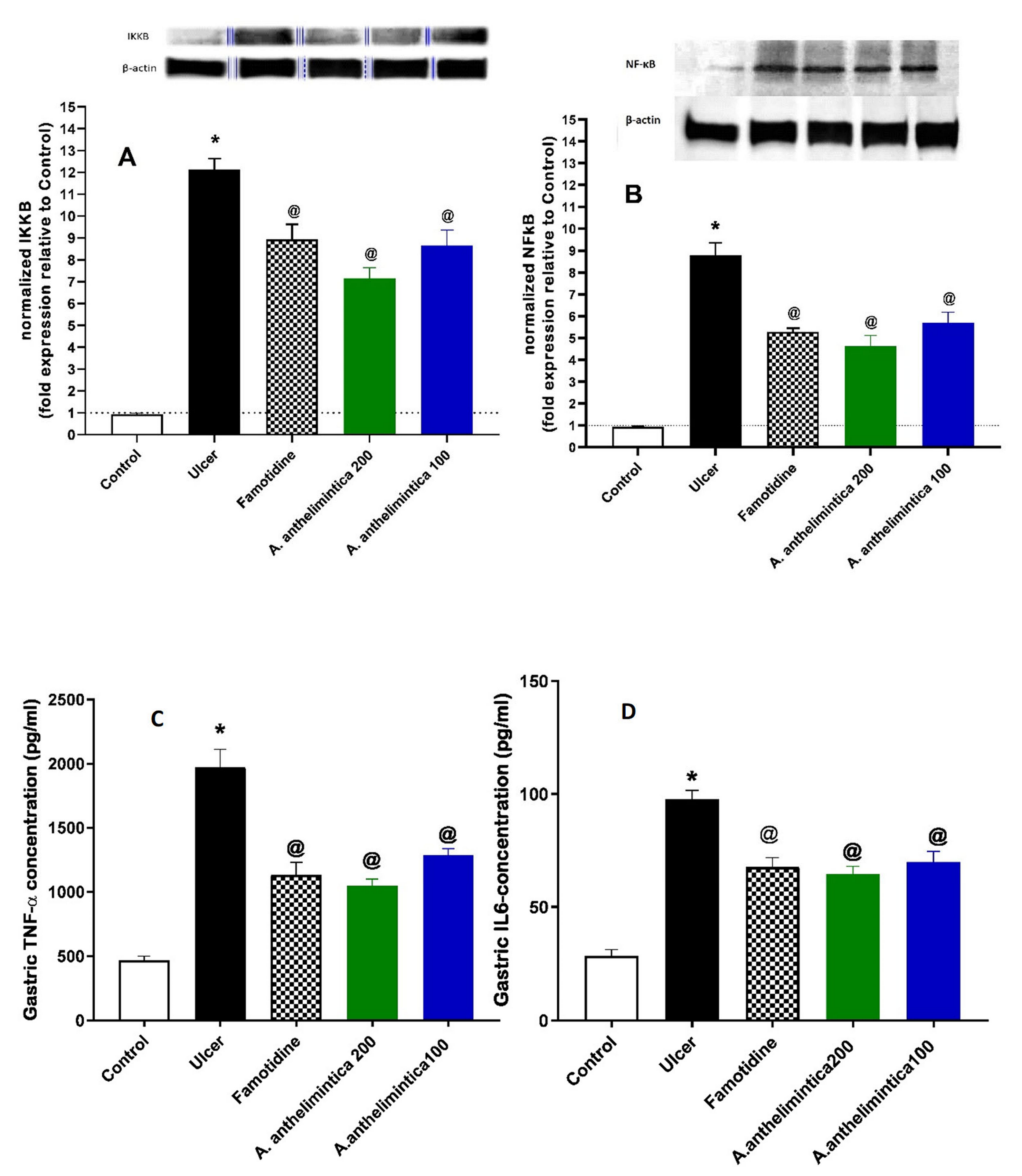
Effects of indomethacin alone and with oral pre-treatments of famotidine or *A. anthelmintica* (200, 100 mg/kg) on (**A**) gastric inhibitor of nuclear factor kappa-B kinase subunit beta (IKKB), (**B**) gastric nuclear factor KB (NF-κB), (**C**) gastric TNF-α, and (**D**) gastric IL-6 in rats. Statistical analyses were carried out by one-way ANOVA followed by Tukey’s post hoc test, mean ± SEM. * Significantly different from control group at *p* < 0.05. ^@^ Significantly different from indomethacin alone group at *p* < 0.05.

Administration of indomethacin caused gastric inflammation, as manifested by marked elevations of the levels of IKKB (9-fold), NF-κB (13-fold), TNF-α (4-fold), and IL-6 (3.4-fold) as compared to the control group (*p* < 0.05). Pre-treatment with famotidine markedly diminished the elevated levels of IKKB NF-κB, TNF-α, and IL-6 by 26.6%, 39.8%, 42.7%, and 30.6%, respectively, as compared to the indomethacin group. Similarly, the high dose of *A. anthelmintica* extract significantly decreased the inflammatory markers by 40.9%, 47.8%, 47%, and 34%, respectively, versus the indomethacin group. The low dose of the extract also significantly decreased the inflammatory markers by 28.2%, 35.2%, 35%, and 28.4% respectively as compared to indomethacin-treated rats (Figure 7).

### 3.8. Effects of A. anthelmintica Extract on Changes in Gastric iNOS Immunoexpression Induced by Indomethacin in Rat Gastric Mucosa

**Figure 8:**
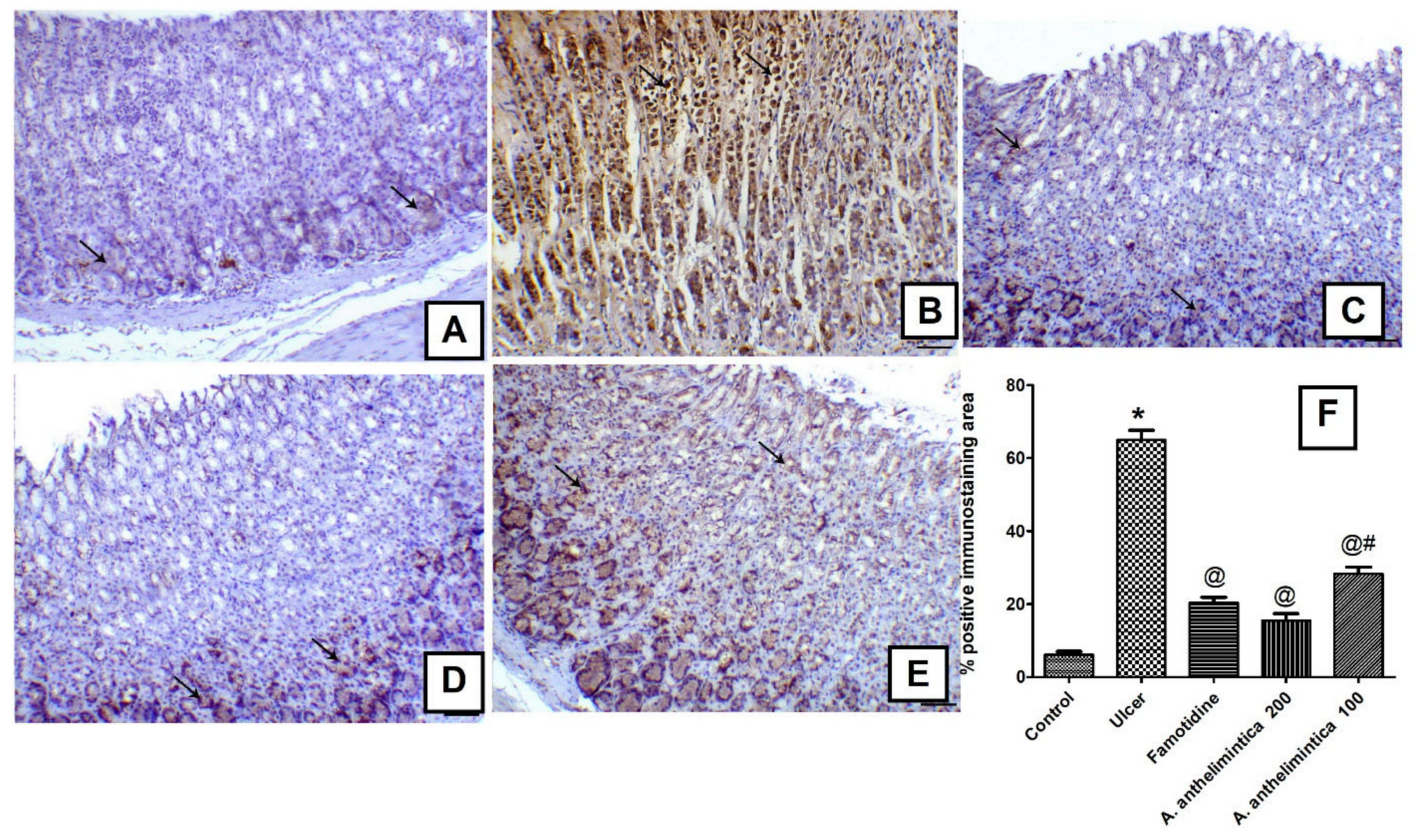
Effects of indomethacin alone and with oral pre-treatments of famotidine or *A. anthelmintica* (200, 100 mg/kg) on gastric inducible nitric oxide expression (iNOS) in rats. (**A**) Gastric mucosa of the control group showing scanty immunoexpression of iNOS within the basal parts of the gastric glands (arrows), (**B**) gastric mucosa of the ulcer group showing marked expression of iNOS within the degenerated gastric glands (arrows), (**C**) gastric mucosa of famotidine group showing marked decrease the expression of iNOS within the gastric glands (arrows), (**D**) gastric mucosa of *A. anthelmintica* (200 mg/kg) group showing marked decrease in the expression of iNOS within the gastric glands (arrows indicate expression within the basal parts of the glands), (**E**) gastric mucosa of *A. anthelmintica* (100 mg/kg) group showing a decrease in the expression of iNOS within the gastric glands (arrows), iNOS antibody, bar = 100 µm. (**F**) Representative bar graph of the percentage of immunopositive area. Statistical analyses were carried out by one-way ANOVA followed by Tukey’s post hoc test, mean ± SEM. * Significantly different from control group at *p* < 0.05. ^@^ Significantly different from indomethacin (ulcer) group at *p* < 0.05. ^#^ Significantly different from *A. anthelmintica* (200 mg/kg) group at *p* < 0.05.

The gastric tissues obtained from the indomethacin-induced gastric ulcer rat model were used for immunohistochemical localization of iNOS. In the indomethacin group, we observed marked expression of iNOS within the degenerated gastric glands compared to the control group, which revealed scanty immunoexpression of iNOS within the basal parts of the gastric glands. Pre-treated rats with different doses of *Albizia* or famotidine showed decreased expression of iNOS within the gastric glands, which was significant when compared to the indomethacin group (*p* < 0.05). The effect of *Albizia* extract on iNOS expression was dose dependent and was higher in the 200 mg/kg group compared to the low-dose group (*p* < 0.05). The high dose’s effect was similar to that of famotidine (Figure 8). 

### 3.9. Molecular Docking Studies

Flavonoids, among them the specific inhibitor naringenin, showed promising inhibition activities against histidine decarboxylase, and therefore, decreased gastric mucosal histamine content [11,26]. In this regard and to explore the molecular mechanism by which the studied plant alleviates the deleterious effects of indomethacin, we virtually screened the possible interactions between the phytoconstituents of the extract and the binding site of the enzyme. All the compounds identified demonstrated moderate binding free energies that ranged between −16.40 and −25.21 kcal/mol; see Table 2. Moreover, plant-derived polyphenols, among them myricetin, inhibited the gastric proton pump and were able to ameliorate gastric acid secretion [27]. They showed moderate free energies of binding towards the gastric proton pump; see Table 2. However, this was not observed in the in vivo studies.

## 4. Discussion

For the first time, we explored the effects of *A. anthelmintica* on indomethacin-induced gastric ulcers in rats. We showed that *A. anthelmintica* at two dose levels (100 and 200 mg/kg) exerted gastroprotective effects comparable in most aspects to the well-known gastroprotective H2- blocker famotidine. Our findings not only showed the gastroprotective effects of *A. anthelmintica*, but also outlined the involved mechanisms of this effect.

The following findings were obtained: (1) The extract ameliorated the structural derangements of the gastric mucosa and improved the ulcer index; the high dose exerted the most potent effects compared to the low dose and famotidine in reversing structural derangements. (2) Both doses increased mucin secretion without reducing gastric acidity. (3) They reduced lipid peroxidation and increased glutathione peroxidase activity, but only the high dose increased the gastric content of reduced glutathione. (4) Besides demonstrating the anti-inflammatory actions of *A. anthelmintica* extract previously, we also showed, for the first time, a modulatory action of *A. anthelmintica* extract on IKKB/NF-KB/TNF-α/iNOS signaling as a contributing mechanism regarding its gastroprotective potential.

Indomethacin induces gastric mucosal lesions by the activation of inflammatory cells and the production of proinflammatory cytokines, and in part by oxidative stress induction [28]. Pre-treatment with *A. anthelmintica* extract improved gastric mucosal damage, as evidenced by a remarkable reduction of the ulcer index with a similar preventive index to that provided by famotidine (the reference drug). In context, our previous work reported the presence of triterpenoid saponins, several flavonoids, and galloyl-glucose derivatives in the extract [13]. Tannins may also prevent ulcer development because of their vasoconstricting and astringent effects. The latter facilitates the microproteins’ precipitation on the ulcer site, forming a layer over the lining that prevents gastric secretions and protects the mucosa from harmful substances and elements [29]. Antioxidant secondary metabolites, among them flavonoids, prevent the formation of gastric lesions caused by ulcerogens and necrotic agents [11,12]. 

Most of the drugs currently used in the treatment of ulcer and gastro-esophageal reflux—such as proton pump inhibitors and H2 blockers, among which our standard reference drug famotidine belongs—act by reducing gastric acid secretion. Acid suppression increases blood levels of gastrin in most patients. Gastrin not only increases gastric acid secretion. but also develops, partially, enterochromaffin-like cell hyperplasia and carcinoids [30]. Furthermore, reduction in gastric acidity increases the risk of enteric infections and changes gastritis distribution in *H. pylori* infected patients. This favors predominant gastritis development with gastric atrophy, which increases the risk for gastric adenocarcinoma and intestinal metaplasia. Moreover, reduction in gastric acidity impairs calcium absorption in the intestine and vitamin B12 absorption, and increases the risk of bone fracture and anemia [31]. We observed that the gastroprotective effects of *A. anthelmintica* extract were not associated with a reduction in gastric acidity. The insignificant increase in the gastric pH caused by the extract might be due to the moderate inhibitory potential of the extract components towards the gastric proton pump and histidine decarboxylase, as revealed from the molecular docking results. This provides the extract an advantage over the existing anti-ulcer drugs.

Previous studies showed that indomethacin administration is associated with increased GI oxidative injury and ROS generation. The peroxidase-catalyzed metabolism of NSAIDs is responsible for the pro-oxidant potential of indomethacin in gastric mucosa [32]. Increased ROS production is associated with increased lipid peroxidation of gastric cell membranes, elevated lactate dehydrogenase leakage and mucosal malondialdehyde, reduced secretion of gastric mucus, and DNA damage [33]. Malondialdehyde (MDA) is considered a biomarker related to lipid peroxidation. Interestingly, in our work, the elevated level of MDA, associated with mucosal injury, was greatly reduced by *A. anthelmintica* extract at both dose levels.

It is well established that the selenium-dependent enzyme glutathione peroxidase (GPx) acts as a defensive barrier against hydroperoxide attack [34]. The GI isoenzyme of GPx activation plays an essential role in increasing the reduced glutathione (GSH) level, which preserves the organism redox status [35]. Like the previous studies [11,33], we showed that indomethacin administration decreased both GPx activity and caused depletion of GSH. The reduced glutathione peroxidase activity aggravated gastric mucosal damage because of the instant elevation of hydrogen peroxide and lipid peroxides in gastric mucosal cells. Therefore, antioxidant enzymes’ upregulation, among them GPx, and elevation of GSH gastric content, could constitute a substantial defense mechanism against GI ulcers associated with oxidative stress. We previously reported that *A. anthelmintica* extract demonstrated potent antioxidant effects both in vitro and in vivo [13]. We also showed that administration of *A. anthelmintica* extract mitigated the disturbed oxidative status associated with gastric injury. Both dose levels increased GPx activity, but only the high dose increased the GSH gastric content, indicating dose-dependent antioxidant activity. The latter effects might be attributed to its high contents of flavonoids and galloyl-glucose derivatives which preserve the gastric GSH level by acting as scavenging free radicals instead of GSH.

The current work proved that gastric injury induction activated the IKκB/NF-κB signal transduction pathway, as displayed by the remarkable increases in gastric mucosal IKKB and NF-κB protein levels compared to the normal mucosa of control rats. *A. anthelmintica* extracts markedly attenuated the IKκB/NF-κB signaling cascade triggered by indomethacin, as manifested by the remarkable decrements in IKκB and NF-κB protein levels compared to the injured mucosa. The extract inhibited the NF-κB activation by preventing the phosphorylation and IκBα subsequent degradation. The down-regulatory effects of *A. anthelmintica* extract on IKκB /NF-κB signaling might be associated with its suppressive effect on IL-6 and TNF-α levels, and antioxidant characteristics [13]. Previous studies showed that antioxidants, among them polyphenolics, hinder NF-κB activation by inhibiting the signal-induced phosphorylation of IκBα [36]. It was also reported that NF-κB inhibition blocks the expression of the pro-inflammatory mediator in gastrointestinal damage, COX-2 [37]. Although in the present study we did not measure COX-2 in gastric mucosa, we previously reported that *A. anthelmintica* inhibited the COX-2 enzyme in vitro, which might also have contributed to the gastroprotective effects of the extract in the current work. In this regard, the suppressive effects of *A. anthelmintica* extract on the level of IKκB/NF-κB signaling might explain the down-regulated expression of COX-2 and the suppression of indomethacin-induced gastric inflammation [13].

Indomethacin caused high-grade inflammation of the gastric mucosa, as manifested by the significant increments in gastric mucosal inflammatory cell infiltration, and elevated TNF-α and IL-6 content, compared to the intact gastric mucosa in control rats. The extract at both dose levels suppressed gastric inflammatory cell infiltration and resulted in remarkable declines in mucosal TNF-α and IL-6 contents, indicating an anti-inflammatory role for *A. anthelmintica*. These results are in context with our previous observation that the extract possesses potent anti-inflammatory potential in the carrageenan-induced hind paw edema model [13]. IL-6 stimulates the lymphocytes, macrophages, and neutrophils at the inflammation site, which leads to the excessive production of ROS and lysosomal enzymes, which in turn cause tissue damage in gastric ulcers. TNF-α plays a major role in indomethacin-induced gastric mucosal injury [38], as it reduces blood flow to the gastric mucosa and increases the gene expression of gastrin and vascular endothelial growth factor in the gastric mucosa, and thus hinders the ulcer healing process [39]. TNF-α also activates the NF-κB pathway that mediates the transcription of several adhesion molecules, leading to the inflammatory cell infiltration detected in indomethacin-treated rats. Additionally, the infiltrating leukocytes represent a major source of ROS generation that would further impair the oxidative status. Notably, *A. anthelmintica* extract, at the two dose levels, ameliorated the inflammatory cell infiltration and significantly mitigated the elevated gastric contents of both TNF-α and IL-6 in indomethacin-treated rats. 

Previous studies showed that TNF-α production could increase nitric oxide (NO) production by iNOS overexpression in indomethacin-induced jejunoileitis [40]. NO produced by inducible nitric oxide synthase (iNOS), in massive amounts, plays an important role in ulcer formation via the formation of peroxynitrite radicals (ONOO-) and cell toxicity, protein tyrosine nitration, hydroxyl radical production, and subsequent tissue damage [11]. The present study showed that indomethacin increased the expression of iNOS in gastric tissues. This may be related to the elevation of TNF-α production in the gastric tissues. The extract exerted dose-dependent inhibition of iNOS expression comparable with that of famotidine, and thus prevented the abundant release of NO that exacerbates gastric mucosal injury, and ultimately, it improved the healing of ulcers.

The current work proved, for the first time, the substantial gastroprotection of *A. anthelmintica* against indomethacin-induced gastric mucosal injury with similar activities to famotidine (The reference drug). It also demonstrated the antioxidant and potent anti-inflammatory effects that are probably mediated by suppressing TNF-α/IKκB/NF-κB /iNOS signaling without reducing gastric acidity, and thus it overcomes the side effects of the chemical antisecretory drugs used in practice. These activities are in agreement with several plant extracts rich in polyphenolics [11,12,41].

## 5. Conclusions

This study demonstrated that gastric ulcers caused by administration of indomethacin were significantly attenuated by *A. anthelmintica* extract. Further, it provides evidence that the therapeutic efficacy of the extract is attributable to the downregulation of the IK*κ*B/NF-*κ*B/TNF-*α/*iNOS signaling pathway and antioxidant effects without reducing gastric acidity. In conclusion, the extract could be considered an effective therapy to treat inflammation and NSAIDs-induced gastric ulcer, while overcoming the adverse effects of traditional antisecretory drugs used in practice.

## Figures and Tables

**Table 1:** Effect of *A. anthelmintica* extract on gastric damage induced by indomethacin in rats.

Groups	Necrosis	Hemorrhage	Edema	Inflammation
Control	0 ± 0	0 ± 0	0 ± 0	0 ± 0
Indomethacin	2.67 * ± 0.21	3.67 * ± 0.21	4.00 * ± 0	3.33 * ± 0.21
Famotidine	1.00 ± 0.0	1.67 ± 0.21	1.67 ± 0.21	1.33 ± 0.21
Extract 200 mg/kg	0.33 ^@^ ± 0.21	0.33 ^@#^ ± 0.21	1.33 ^@^ ± 0.21	1.00 ^@^ ± 0
Extract 100 mg/kg	1.67 ± 0.21	2.00 ± 0	2.67 ± 0.21	2.00 ± 0

Data are presented as mean ± SEM, *n* = 6. Statistical analyses were carried out using Kruskal–Wallis tests followed by Dunns’ post hoc test. * Significantly different from control group at *p* < 0.05. ^@^ Significantly different from indomethacin group at *p* < 0.05. ^#^ Significantly different from famotidine and extract (100 mg/kg) groups at *p* < 0.05.

**Table 2:** Scoring functions and amino acid interactions for the phytoconstituents from *A. anthelmintica* docked to histidine decarboxylase and gastric proton pump.

Compounds ^#^	Histidine Decarboxylase (4E1O)		Gastric Proton Pump (5YLV)	
	Scoring Function	Amino Acid Residues Interactions	Scoring Function	Amino Acid Residues Interactions
Galloyglucose	−18.15	SER 196 (H-bonding) HIS 194 (H-bonding, Hydrophobic)	−12.27	THR 134 (H-bonding) GLU 900 (H-bonding) LEU 811 (H-bonding)
Hydroxybenzyl*-O-*galloyl-glucose	−24.86	GLY 246 (H-bonding) ASP 273 (H-bonding) SER 151 (H-bonding)	−16.81	LEU 811 (H-bonding) TYR 799 (Hydrophobic)
Pyrogallol*-O-*methylgalloyl-glucose	−24.53	SER 196 (H-bonding) ASP 273 (H-bonding) TYR 80 (Hydrophobic)	−18.06	GLU 900 (H-bonding) ASP 137 (H-bonding) ASN 138 (H-bonding)
Glyceric acid*-O-*galloy glucose	−20.42	PRO 82 (H-bonding) SER 151 (H-bonding) SER 196 (H-bonding) HIS 194 (H-bonding, Hydrophobic)	−15.08	THR 134 (H-bonding) GLU 900 (H-bonding) LEU 811 (H-bonding) ASN 138 (H-bonding)
Coumaroyl*-O-*galloyl-glucose	−23.13	SER 151 (H-bonding) ASP 273 (H-bonding) GLY 246 (H-bonding)	−13.52	GLU 900 (H-bonding) LEU 811 (H-bonding) GLN 127 (H-bonding) TYR 799 (Hydrophobic)
Benzyl*-O-*galloyl-glucose	−22.01	SER 151 (H-bonding) ASP 273 (H-bonding)	−17.11	LEU 811 (H-bonding) GLN 127 (H-bonding)
Quercetin glucoside	−24.05	SER 196 (H-bonding) HIS 194 (Hydrophobic) TYR 81 (H-bonding)	−16.78	LEU 811 (H-bonding) ASP 137 (H-bonding) THR 134 (H-bonding)
Quercetin*-O-*galloyl-glucoside	−21.03	GLY 246 (H-bonding) ASP 273 (H-bonding) TYR 81 (H-bonding)	−20.59	LEU 811 (H-bonding) ASP 137 (H-bonding) GLN 127 (H-bonding) ASN 138 (H-bonding)
Quercetin benzoyl-glucoside	−20.79	GLY 246 (H-bonding) ASP 273 (H-bonding) TYR 81 (H-bonding) HIS 194 (Hydrophobic)	−18.51	GLU 900 (H-bonding) LEU 811 (H-bonding) GLN 127 (H-bonding) ASN 138 (H-bonding) THR 134 (H-bonding)
Quercetin coumaroyl-glucoside	−19.59	GLY 246 (H-bonding) TYR 81 (H-bonding) ASP 273 (H-bonding) HIS 194 (H-bonding, Hydrophobic)	−17.83	LEU 811 (H-bonding) GLN 127 (H-bonding) LEU 133 (H-bonding)
Quercetin caffeoyl-glucoside	−24.41	SER 151 (H-bonding) TYR 81 (H-bonding) GLY 246 (H-bonding)	−21.58	GLU 900 (H-bonding) LEU 811 (H-bonding) LEU 811 (H-bonding)
Kaempferol glucoside	−24.06	PRO 82 (H-bonding) GLY 246 (H-bonding) ASP 273 (H-bonding) HIS 194 (Hydrophobic)	−16.44	LEU 811 (H-bonding) GLU 343 (H-bonding) GLU 795 (H-bonding) CYS 813 (H-bonding)
Kaempferol galloyl-glucoside	−25.21	SER 196 (H-bonding) THR 248 (H-bonding) TYR 81 (H-bonding) ASP 273 (H-bonding)	−19.67	LEU 811 (H-bonding) GLU 900 (H-bonding) GLN 127 (H-bonding) LEU 133 (H-bonding) ASP 132 (H-bonding)
Kaempferol coumaroyl-glucoside	−16.40	GLY 246 (H-bonding) ASP 273 (H-bonding) TYR 81 (H-bonding) HIS 194 (H-bonding) SER 151 (Hydrophobic) HIS 194 (Hydrophobic)	−18.30	GLU 900 (H-bonding) GLN 127 (H-bonding)

^#^ Previously described from the plant in Sobeh et al. [13].
